# Dr. Joseph Allen Osmun, Jr.

**Published:** 1917-03

**Authors:** 


**Dr. Joseph Allen Osmun, Jr.,** Buffalo 1916, died at the Buffalo General Hospital, of pneumonia, Feb. 8, after an illness of less than a week. He was born in Newark, N. J., 1894 but moved to Whittier, Calif., some years ago, with his parents and graduated from Leland Stanford Jr. University. While studying at the University of Buffalo, he joined the 65th Reg. and went with this after its conversion into the 3d Artillery, to Camp Whitman. He was injured there, so as to be unable to accompany the regiment to Texas, and returned to Buffalo. He is survived by his father, also a physician. The remains were taken to Newark for burial.

